# Corrigendum to “Inorganic nitrate and nitrite ameliorate kidney fibrosis by restoring lipid metabolism via dual regulation of AMP-activated protein kinase and the AKT-PGC1α pathway” [Redox Biol. 51, (2022), 102266]

**DOI:** 10.1016/j.redox.2026.104413

**Published:** 2026-09-28

**Authors:** Xuechen Li, Zhengbing Zhuge, Lucas Rannier R.A. Carvalho, Valdir A. Braga, Ricardo Barbosa Lucena, Shuijie Li, Tomas A. Schiffer, Huirong Han, Eddie Weitzberg, Jon O. Lundberg, Mattias Carlström

**Affiliations:** a Beijing Key Laboratory of New Drug Mechanisms and Pharmacological Evaluation Study, Institute of Materia Medica, Chinese Academy of Medical Science & Peking Union Medical College, Beijing, China; b Department of Physiology and Pharmacology, Karolinska Institutet, Stockholm, Sweden; c Department of Biotechnology, Federal University of Paraíba, João Pessoa, PB, Brazil; d Department of Veterinary Sciences, Federal University of Paraíba, Areia, PB, Brazil; e Department of Microbiology and Tumor and Cell Biology, Karolinska Institutet, Stockholm, Sweden; f Department of Perioperative Medicine and Intensive Care, Karolinska University Hospital, Stockholm, Sweden

This Corrigendum relates to the above article.

Post-publication, an investigation identified a concern around the authenticity of the following figures/image panels:

- Fig. 6 Panel C (p-AMPK row) and Fig. 8 Panel E (p-AMPK row) appear to depict the same source image, but are described as representing different experimental samples.
- Fig. 6 Panel C (AMPK row) and Fig. 8 Panel E (AMPK row) appear to depict the same source image, but are described as representing different experimental samples.
- Fig. 6 Panel C (Bottom Vinculin row) and Fig. 8 Panel E (Vinculin row) appear to depict the same source image, but are described as representing different experimental samples.

The authors were requested to provide comment on these concerns, as well as the original unprocessed image files to aid investigation, and have clarified that the p-AMPK and AMPK images in Fig. 6C were correctly presented. However, the p-AMPK and AMPK images in Fig. 8E were inadvertently misinserted from Fig. 6C dataset during figure assembly, due to similar file names and storage in the same folder. The correct p-AMPK and AMPK images for Fig. 8E have now been provided as replacements. Notably, the densitometric quantification was performed using the correct source images. Therefore, the quantitative analysis, statistical results, and conclusions remain unchanged. Regarding the Vinculin bands shown in Figs. 6C and 8E, although they appear visually similar, the authors have re-examined the original uncropped Western blot images and laboratory records, and confirmed that they derive from separate, independent experiments representing different samples. Their similar appearance reflects comparable loading patterns and exposure conditions rather than duplication of the same image. The authors have provided the correct figures as below.

**Figure:**
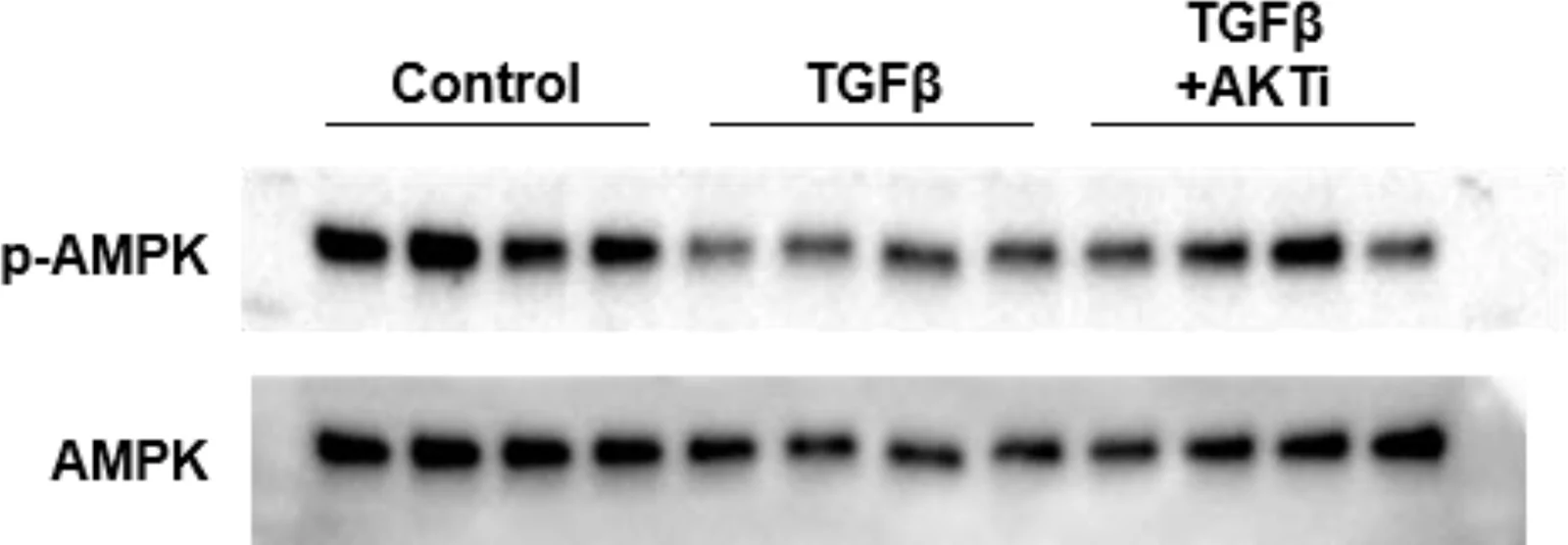

Correct p-AMPK and AMPK bands in Fig. 8E.

The authors apologise for this oversight.

